# Nomogram and scoring system for preoperative prediction of the risk of systemic inflammatory response syndrome in one-stage flexible ureteroscopy lithotripsy

**DOI:** 10.3389/fsurg.2025.1592507

**Published:** 2025-05-09

**Authors:** Yuan Zhou, Haiyan Zhang, Rentao Zhang, Yinman Ding, Zhengquan Wang, Changming Lin

**Affiliations:** ^1^ Department of Urology Surgery, The People’s Hospital of Xuancheng City, Xuancheng, China; ^2^ Wannan Medical College, Wuhu, China; ^3^ Department of Urology Surgery, Huaian 82 Hospital, Huaian, China

**Keywords:** ureteroscopy lithotripsy, upper urinary tract stone, systemic inflammatory response syndrome, nomogram, systemic immune-inflammation index, infection prevention

## Abstract

**Background:**

Flexible ureteroscopy lithotripsy (FURL) is a prevalent intervention for the management of upper urinary tract stones (UUTS). Assessing the onset of systemic inflammatory response syndrome (SIRS) in patients during and postoperatively is a critical determinant in the decision-making process regarding the necessity of preoperative ureteral stenting prior to FURL.

**Materials and methods:**

A total of 340 patients with UUTS who underwent one-stage FURL were analyzed retrospectively. Least absolute shrinkage and selection operator and multivariate logistic regression analysis were used to screen out independent risk factors, subsequently developing a nomogram. The predictive performance was internally assessed using the concordance index (C-index), receiver operating characteristic curve, and calibration curve. Additionally, we evaluated the risk of SIRS in the context of one-stage FURL, considering the impact of various available variables.

**Results:**

Age, urinary white blood cells, urine bacterial culture, and systemic immune-inflammation index (SII) were integrated to establish a nomogram for prediction of the risk of SIRS in patients undergoing one-stage FURL. The SII exhibited the highest odds ratio (OR = 30.356) for SIRS. The nomogram demonstrated a favorable predictive performance with a C-index of 0.964 (95% CI = 0.932–0.996), an area under the curve of 0.935, and a calibration curve validating its accuracy. We further developed a scoring system and classified the risk of SIRS into four grades.

**Conclusion:**

The developed nomogram and risk scoring system demonstrate favorable predictive ability and clinical serviceability for the personalized estimation of SIRS risk in UUTS patients undergoing one-stage FURL. It is advisable to place a ureteral stent prior to FURL in individuals with an SII exceeding 1,300 and meeting one of the following criteria: age > 60 years, urinary white blood cell levels of 1+/2+/3+, or positive urine bacterial culture. The insights provided may assist clinicians in selecting safer therapeutic approaches for UUTS patients.

## 1 Introduction

Upper urinary tract stone (UUTS) is one of the most common diseases in urology, with an incidence rate of approximately 8.8% in the United States, and approximately 25% of patients with UUTS necessitate surgical intervention (1). Flexible ureteroscopy lithotripsy (FURL) is the preferred modality for managing UUTS measuring <2 cm in diameter, owing to its minimally invasive nature and expedited recovery compared with percutaneous nephrolithotomy (PCNL) and open surgical procedures (2). The standard treatment for FURL involves the initial placement of a ureteral stent, followed by lithotripsy in a subsequent stage. Preoperative insertion of a stent 2 weeks prior to FURL has been associated with reduced operative duration, improved stone-free rates, and lower complication rates compared with cases without ureteral stent placement (3). However, the two-stage FURL prolongs the hospitalization time and increases the economic burden on patients. Recently, one-stage FURL has demonstrated superior lithotripsy efficacy and has emerged as the preferred option among clinicians in practice (4, 5). Sepsis represents one of the most serious complications associated with FURL, with systemic inflammatory response syndrome (SIRS) identified as an early indicator of sepsis (3). Assessing SIRS development during and following FURL is a crucial factor in preoperative ureteral stent placement decision. Consequently, this study aimed to develop an effective prediction tool for assessing the risk of SIRS preoperatively in patients undergoing one-stage FURL based on the individual situation and routine laboratory results of UUTS patients.

## 2 Materials and methods

### 2.1 Study population

We conducted a retrospective analysis of patients diagnosed with UUTS who underwent one-stage FURL at the Department of Urology, Xuancheng People's Hospital, between July 2021 and May 2024. All patients presented with UUTS measuring <2.5 cm in diameter and were deemed appropriate candidates for one-stage FURL intervention. Each procedure was performed under general anesthesia. We set the following inclusion criteria in this study: (a) all patients who had confirmed unilateral UUTC via computed tomography and (b) those over 18 years old and who did not have a nephrostomy tube or ureteral stent. The cohort consisted of individuals with unilateral UUTS, mainly ureteral calculi, with no infection beyond the urinary tract. We excluded individuals with significant cardiopulmonary impairment, coagulation abnormalities, or a history of psychiatric disorders, hematologic diseases, immunological disorders, splenic anomalies, and malignant neoplasms. None of the patients had received immunosuppressive agents, hormonal treatments, or antiplatelet medications in the past month. In addition, we excluded patients with solitary kidneys, polycystic kidney disease, horseshoe kidneys, uremia, or preoperative SIRS response. The study population did not include pregnant women or individuals who had undergone blood transfusions or experienced severe hemorrhage within the past year. All surgical procedures were performed by the same surgical team. In addition to the aforementioned exclusion criteria, no other patients who underwent one-stage FURL were excluded from the study.

### 2.2 Data acquisition

We obtained the available information of UUTS patients including age, gender, height, weight, stone site, stone size, diabetes, hydronephrosis, neutrophils, lymphocytes, platelets, blood creatinine, urinary white blood cells (WBC), urine bacterial culture, ureteral access sheath, and surgery time. For patients with urine WBC levels of 2+/3+ or positive urine cultures, a course of second-generation cephalosporin or sensitive antibiotic was administered for anti-inflammatory treatment 2 days before surgery. All patients received second-generation cephalosporin or sensitive antibiotic 30 min before FURL to prevent the risk of infection. Mild hydronephrosis indicates a separation of the renal pelvis <2 cm, whereas moderate or severe hydronephrosis is associated with a renal pelvis dilation exceeding 2 cm. The body mass index (BMI) value is determined by an individual's height and weight measurements. The estimated glomerular filtration rate (EGFR) value is calculated based on the patient's gender, age, and is is is determined based on serum creatinine levels. The systemic immune-inflammation index (SII) is derived from the counts of blood neutrophils, lymphocytes, and platelets. A one-stage FURL signifies that patients underwent the procedure without prior placement of a ureteral stent. A two-stage FURL indicates that ureteral stenting was performed prior to the FURL. All patient examination information was preoperative results. SIRS positivity is defined as meeting at least two of the following criteria: body temperature exceeding 38.5°C or falling below 36°C; heart rate >90 beats per minute; respiratory rate exceeding 20 breaths per minute or PaCO_2_ below 32 mmHg; peripheral blood leukocyte count >12 × 10^9^/L or <4 × 10^9^/L; or the presence of immature granulocytes exceeding 10% (2). Postoperatively, all patients underwent hematological assessments, and respiratory and heart rates were monitored at least three times daily. If there was no SIRS response observed prior to discharge (typically 3 days postsurgery), the SIRS response was classified as negative.

### 2.3 Statistical analysis

The analysis encompassed various variables, including age, gender, BMI value, diabetes, stone site, stone size, hydronephrosis, urinary WBC, urine bacterial culture, EGFR value, SII value, ureteral access sheath, and surgery time. We used the least absolute shrinkage and selection operator (LASSO) regression to select predictive factors with non-zero coefficients. Subsequently, multivariate logistic regression analysis was conducted to isolate independent risk factors with a *P*-value of <0.05. Variance inflation factor (VIF) analysis was utilized to evaluate the collinearity information among the clinical variables and the final radiomics features. Combined with independent risk factors, we developed a nomogram to personalize the prediction of the risk of SIRS in one-stage FURL. The Hosmer–Lemeshow test was performed to evaluate the goodness-of-fit of the nomogram. The predictive ability of the nomogram was internally evaluated by the receiver operating characteristic (ROC) curve and calibration curve. The concordance index (C-index) was measured to quantify the discrimination performance of the nonadherence nomogram. We used bootstrapping validation, comprising 1,000 bootstrap resamples, which was implemented for the non-adherence nomogram. Additionally, decision curve analysis (DCA) was conducted to test the clinical applicability of the non-adherence nomogram. This analysis was achieved by quantifying the net benefits at various threshold probabilities. The net benefit was computed as the proportion of true-positive patients minus the proportion of false-positive patients, while also taking into account the relative harm of not implementing an intervention as compared with the negative outcomes resulting from an unnecessary intervention. Utilizing the outcomes from each variable subgroup within the nomogram, we developed an SIRS scoring system through mathematical calculation. Subsequently, we divided the SIRS risk into four grades based on the scoring and patient population and compared the SIRS risk across varying grades. All statistical analyses and graphics were performed using R software (version 4.4.1), and a two-tailed *P*-value of <0.05 was deemed statistically significant.

## 3 Results

### 3.1 Patient data

A total of 340 UUTS patients who underwent one-stage FURL were selected in this study, of whom 31 (9.12%) developed SIRS. We compared age, gender, BMI value, diabetes, stone site, stone size, hydronephrosis, urinary WBC, urine bacterial culture, EGFR value, SII value, ureteral access sheath, and surgery time between the non-SIRS group and SIRS group. The baseline characteristics of both groups are listed in Table 1.

**Table 1:** Baseline characteristics of the study cohort (*n* = 340).

Characteristics	Non-SIRS group (*n* = 309)	SIRS group (*n* = 31)	*χ*^2^ value	*P*-value
	*N* (%)	*N* (%)		
Gender			6.200	0.013
Male	217 (70.23%)	15 (48.39%)		
Female	92 (29.77%)	16 (51.61%)		
Age (years)			3.679	0.055
≤60	261 (84.47%)	22 (70.97%)		
>60	48 (15.53%)	9 (29.03%)		
Stone site			3.825	0.050
Left	173 (55.99%)	23 (74.19%)		
Right	136 (44.01%)	8 (25.81%)		
Stone size (cm)			0.763	0.382
<0.8	155 (50.16%)	13 (41.94%)		
≥0.8	154 (49.84%)	18 (58.06%)		
Ureteral access sheath			0.295	0.863
F10–12	89 (28.80%)	8 (25.81%)		
F11–13	115 (37.22%)	11 (35.48%)		
F12–14	105 (33.98%)	12 (38.71%)		
Hydronephrosis			0.698	0.404
Mild	239 (77.35%)	26 (83.87%)		
Moderate or severe	70 (22.65%)	5 (16.13%)		
Urinary white blood cell			79.226	<0.001
None or weakly positive	200 (64.72%)	7 (22.58%)		
1+ or 2+	95 (30.75%)	8 (25.81%)		
3+	14 (4.53%)	16 (51.61%)		
Urine bacterial culture			57.741	<0.001
None	303 (98.06%)	21 (67.74%)		
Yes	6 (1.94%)	10 (32.26%)		
Diabetes			0.141	0.708
None	285 (92.23%)	28 (90.32%)		
Yes	24 (7.77%)	3 (9.68%)		
Surgery time (hours)			5.268	0.022
<1	267 (86.41%)	22 (70.97%)		
≥1	42 (13.59%)	9 (29.03%)		
BMI value (kg/m^2^)			0.078	0.780
<22.9	102 (33.01%)	11 (35.48%)		
≥22.9	207 (66.99%)	20 (64.52%)		
EGFR (ml/min/1.73 m^2^)			2.397	0.122
<90	125 (40.45%)	17 (54.84%)		
≥90	184 (59.55%)	14 (45.16%)		
SII value			141.436	<0.001
<700	225 (72.82%)	1 (3.23%)		
700–1,300	67 (21.68%)	6 (19.35%)		
>1,300	17 (5.50%)	24 (77.42%)		

*χ*^2^ is chi-square.

EGFR, estimated glomerular filtration rate; BMI, body mass index; SII, systemic immune-inflammation index.

### 3.2 Independent risk factors

**Figure 1:**
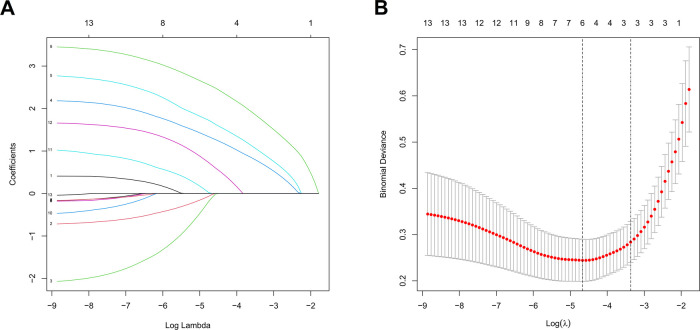
LASSO regression to screen out optimal parameter factors **(A)** and the relationship between each clinical variable and log (*λ*) after parameter adjustment **(B)** in the LASSO model, 10-fold cross-validation was utilized to determine the optimal parameter *λ* for the selection of radiomic features. At the optimal tuning parameter *λ* = 0.012 and log (*λ*) = −1.907, the dotted vertical lines were placed at the optimal values determined by the minimum criteria. A coefficient profile plot was created with respect to the log (*λ*) sequence. The dotted vertical lines were drawn corresponding to the six non-zero coefficients, indicating the optimal value of *λ* in the least absolute shrinkage and selection operator.

The feature selection of LASSO regression showed 13 features which were reduced to 6 potential predictors (Figure 1), including age, stone site, hydronephrosis, urinary WBC, urine bacterial culture, and SII value. These non-zero coefficient predictors were subjected to further investigation through multivariate logistic regression analysis, and variables with *P*-values of <0.05 were considered independent risk factors. VIFs of the six radiomics features were tolerable (VIF value for variables: age = 1.062, stone site = 1.041, hydronephrosis = 1.276, urinary white blood cell = 1.228, urine bacterial culture = 1.218, SII = 1.129). The independent risk factors including age, urinary WBC, urine bacterial culture and SII value, and SII exhibited the highest odds ratio (OR) (value = 30.356; 95% CI = 10.215–142.17) in relation to SIRS, followed by urine bacterial culture (Table 2).

**Table 2:** Multivariate logistic regression analysis of prediction factors for the study cohort.

Characteristics	*β*	SE	Odds ratio (95% CI)	*P*-value
Age	1.691	0.760	5.423 (1.253–25.971)	0.026
Stone site	−0.787	0.727	0.455 (0.099–1.796)	0.279
Hydronephrosis	−1.667	1.119	0.189 (0.016–1.418)	0.136
Urinary white blood cell	1.943	0.502	6.979 (2.853–21.063)	<0.001
Urine bacterial culture	2.711	1.278	15.037 (1.456–227.307)	0.034
SII	3.413	0.656	30.356 (10.215–142.17)	<0.001

*β* is the regression coefficient. SE is the standard error.

CI, confidence interval; SII, systemic immune-inflammation index.

### 3.3 Establishment and validation nomogram

**Figure 2:**
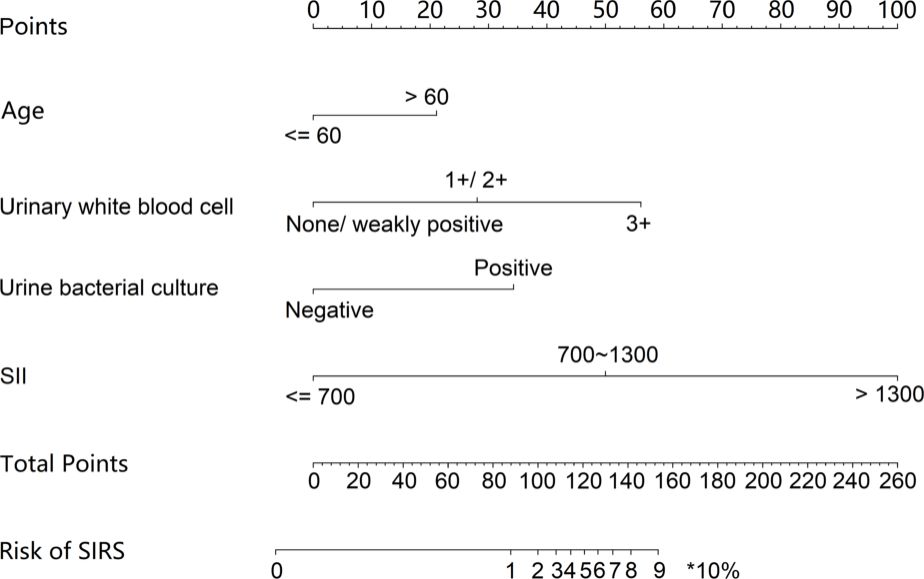
Nomograms for preoperative prediction of the risk of SIRS in patients undergoing one-stage FURL.

**Figure 3:**
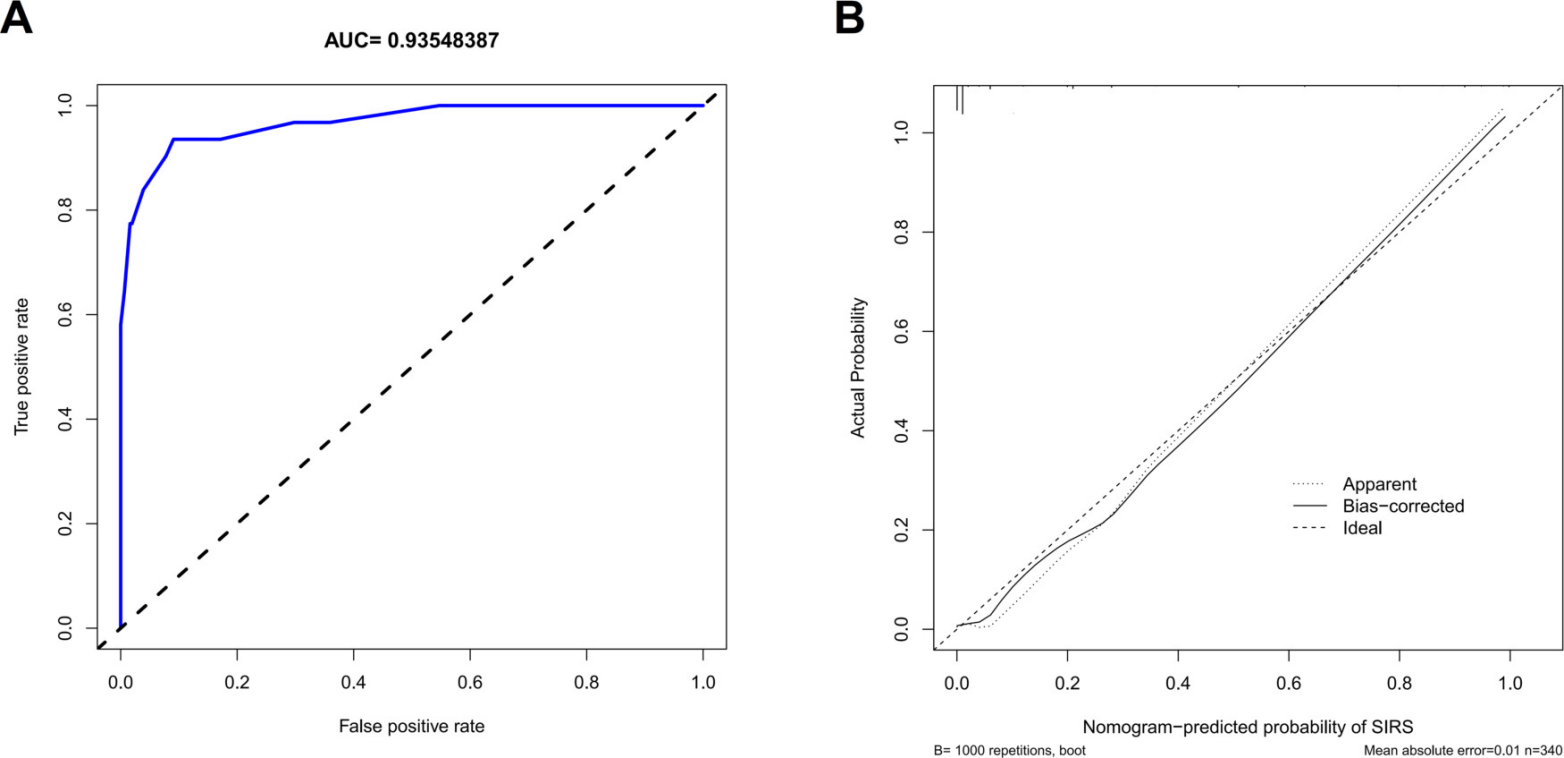
Receiver operating characteristic curve **(A)** and calibration curve **(B)** to internally verify the predictive accuracy of nomogram prediction.

**Figure 4:**
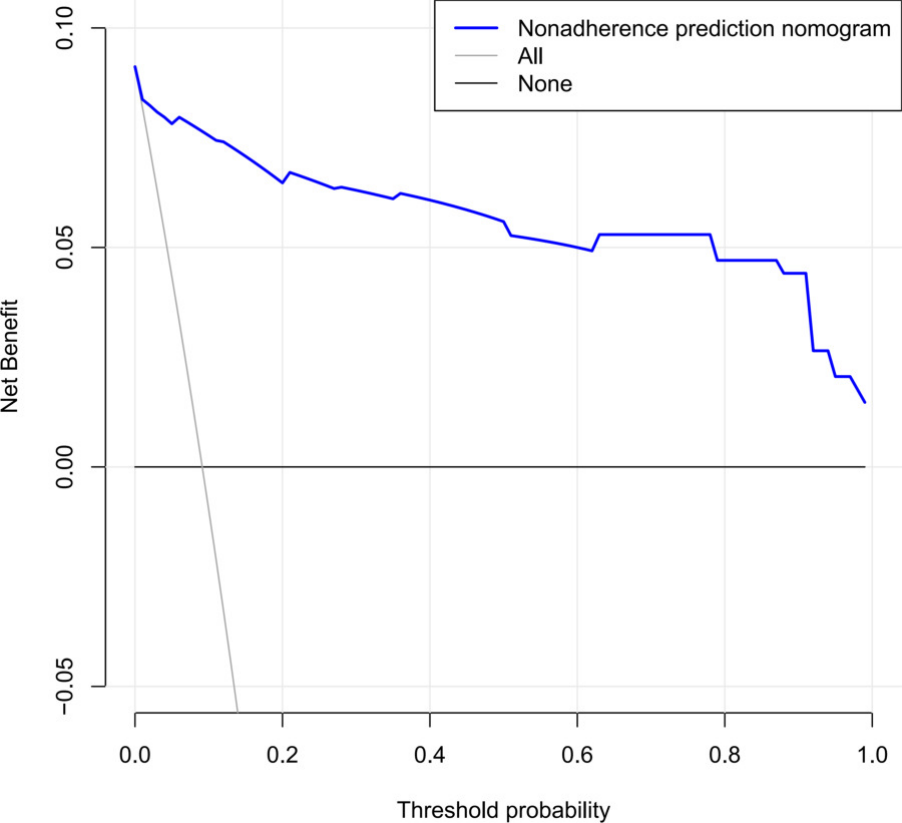
Decision curve analysis to test the clinical serviceability of the nomogram.

The independent risk factors, including age, urinary WBC, urine bacterial culture, and SII value, were integrated to develop a nomogram for individualized risk assessment of SIRS in patients undergoing one-stage FURL (Figure 2). Calibration was evaluated using the Hosmer–Lemeshow test with adaptive grouping (group = 5), resulting in a chi-square statistic of 2.507 (*P* = 0.474), which indicates no significant deviation between the predicted probabilities and observed outcomes, thereby suggesting robust calibration of the nomogram. The concordance index (C-index) was calculated at 0.964 (95% CI = 0.932–0.996), and the area under the curve (AUC) was determined to be 0.935, both nearing perfect performance. The calibration curves of the non-adherence nomogram indicated a favorable predictive capability for estimating the risk of SIRS in one-stage FURL (Figure 3). The DCA curve showed a net benefit within almost all threshold probability ranges of patients in this study, demonstrating the clinical effectiveness of our prediction model (Figure 4).

### 3.4 Risk scoring system for SIRS

By integrating the subgroup scores presented in the nomogram, we developed a risk scoring system to quantify the risk of SIRS for one-stage FURL. This risk scoring system was structured with a maximum of 100 points, wherein the higher score indicated a greater risk of SIRS occurrence in one-stage FURL (Table 3). The potential for SIRS development was classified into four grades, based on the subgroup scores and the number of patients with UUTC. The incidence and risk multiples for SIRS at each grade were shown in Table 4, indicating that 82.76% of patients classified in Grade IV developed SIRS, presenting a risk multiplier of 9.077 compared with the general cohort of UUTS patients (Table 4).

**Table 3:** Scores of each subgroup in the risk scoring system.

Characteristics	Points	Characteristics	Points
Age (years)		Urine bacterial culture	
≤60	0	Negative	0
>60	10	Positive	17
Urinary white blood cell		SII value	
None/weakly positive	0	≤700	0
1+/2+	13	700–1,300	24
3+	26	>1,300	47

SII, systemic immune-inflammation index.

**Table 4:** The comparison of each grade in the risk scoring system.

Grade	Points	Number (%)	Number of patients with SIRS	Incidence rate (%)	Risk multiples
I	0–20	202 (59.41%)	1	0.50%	0.054
II	21–40	79 (23.24%)	1	1.267%	0.134
III	41–55	30 (8.82%)	5	16.67%	1.828
IV	56∼100	29 (8.53%)	24	82.76%	9.077

## 4 Discussion

Patients undergoing one-stage FULR exhibit an elevated incidence of SIRS compared with those receiving two-stage FULR (3). Indications for stent placement prior to FURL may include the presence of obstructive ureteral stones, calculus-induced pyelonephritis, patient-reported pain, or the need to facilitate the insertion of the flexible ureteroscope sheath (3). Our investigation encompassed 340 UUTS patients who received one-stage FURL, with 31 individuals (9.12%) developing SIRS. Previous research has indicated that the incidence of SIRS during and after FURL ranges from 6.7% to 20.7% (6). Sepsis represents the most severe complication associated with FURL, potentially resulting in septic shock and mortality (7). Levy et al. (8) compiled data on 25,375 sepsis patients, including 18,766 from the United States and 6,609 from Europe, reporting adjusted mortality rates of 32.3% and 31.3%, respectively. Therefore, urologists need to manage the risk of SIRS associated with FURL through general preoperative examinations and convenient tools.

In this study, we selected SIRS as an indicator to reflect urinary tract infections in UUTS patients. SIRS is a widely recognized biomarker of systemic inflammation and is recognized as an early manifestation of sepsis (9–11). Some scholars have used other methodologies for the early prediction of sepsis, such as quick sequential organ failure assessment (qSOFA), which predicts sepsis risk based on changes in respiratory rate, systolic blood pressure, and mental status. However, its predictive validity has been suboptimal (12, 13). A statistical analysis conducted by Norwegian researchers on a cohort of 1,535 infected patients revealed that 108 had sepsis, yet only 33 (30.1%) satisfied the qSOFA criteria. Furthermore, among the 26 patients who died within 7 days, merely 4 (15.4%) exhibited a qSOFA score ≥ 2 (12). Some other diseases may lead to false positives in the qSOFA assessment, such as cardiac embolism, respiratory distress syndrome, severe trauma, and anaphylactic shock. Due to the limited sensitivity of qSOFA to sepsis, international sepsis and septic shock management guidelines strongly advise against its use as a standalone screening instrument and emphasize that SIRS is more effective in assessing sepsis severity compared with qSOFA, blood lactate, and other biomarkers (13).

The variables in our study were derived from routine preoperative examinations of patients with UUTS, and the result showed that age, urinary WBC, urine bacterial culture, and SII value were identified as independent risk factors for the development of SIRS. In contrast to younger UUTS patients, older patients exhibited diminished immune functionality and a higher prevalence of potential diseases, thereby increasing their susceptibility to SIRS. In our analysis, a urine WBC 3+ was associated with a sensitivity of 0.53 and a specificity of 0.95 in predicting SIRS, while the sensitivity and specificity for SIRS prediction based on urine bacterial culture were found to be 0.63 and 0.94, respectively. Previous studies utilizing multiple logistic regression analysis had found that positivity for urine WBC, urine bacterial culture, and urine nitrite were independent risk factors for the onset of fever and SIRS in patients with UUTC (1, 14). Nitrite is usually not present in normal urine. Urinary bacteria, particularly gram-negative species, can convert nitrate into nitrite. In this study, only six patients exhibited nitrite positivity in their urine, which precluded its inclusion in the analysis. Our results showed that SII had the highest OR (30.356) for the development of SIRS in UUTS patients undergoing one-stage FURL. The sensitivity and specificity of predicting SIRS using urine SII > 1,300 were found to be 0.59 and 0.98, respectively. Among the participants, five patients eventually progressed to sepsis, none died, and all patients with sepsis had an SII exceeding 1,300, with an average SII of 6,750.32. The SII has been proven to be a promising prognostic biomarker for evaluating the development of various inflammatory diseases and the prognosis of different malignancies (15–18). Preoperative SII has also been demonstrated as a prognostic marker for evaluating infection risk in patients undergoing various surgeries, such as knee arthroplasty, cardiac stent implantation, and lung surgery (19–21). Wang et al. (22) found that among the various predictive indicators for early severe mycoplasma pneumoniae pneumonia, the predictive capability of SII was significantly superior to that of neutrophil-to-lymphocyte ratio, platelet-to-lymphocyte ratio, and systemic immune response index, which demonstrates the superiority of SII as an innovative confirmatory predictor in reflecting systemic inflammation. In the field of urological lithotripsy, Peng et al. (23) investigated the role of preoperative SII in forecasting SIRS in PCNL and found that SII exhibited a superior predictive capacity than traditional inflammatory markers. Infectious UUTSs are more prone to SIRS response, where increased neutrophil counts and reduced lymphocyte levels may release pro-inflammatory mediators that could facilitate stone formation. Platelet activation can release vascular permeability factors by releasing 5-hydroxytryptamine from alpha particles and dense particles, which enhance vascular permeability, promote the chemotaxis of leukocytes, and participate in the inflammatory process (23). However, we have not found any studies focusing on preoperative SII as a predictor of infection in the context of FURL. Some other factors, such as pyuria and stone culture, have been verified to be related to the risk of SIRS in stone surgery (24, 25). Nevertheless, the results of these indicators can only be obtained after surgery. The objective of this study was to preliminarily assess the SIRS associated with one-stage FURL through preoperative examination; hence, these indicators were not analyzed.

Nomogram, a statistical tool that graphically represents multiple independent risk factors, is widely utilized to assess the risk of disease incidence and prognosis (26, 27). In contrast to previous nomograms designed to predict SIRS risk in patients undergoing FURL (28, 29), our nomogram model exhibits several distinctions. Firstly, the cohort in this study comprised exclusively patients undergoing one-stage FURL. The focus of this study was to evaluate the risk of SIRS in UUTS patients who did not have ureteral stents placed prior to FURL, thereby enhancing the selection of surgical approaches in clinical practice. Secondly, we are the first to evaluate SII in relation to the development of SIRS in patients undergoing one-stage FURL. Thirdly, we developed a novel risk scoring system for predicting SIRS in one-stage FURL, categorizing the risk into four grades. Within our risk scoring system, SII > 1,300 had the highest score of 47. Our risk scoring system showed Grade IV with points 55–100, and the risk multiple for developing SIRS in Grade IV was 9.077 compared with the general population. We strongly advise that UUTS patients with a preoperative score of Grade IV should undergo a two-stage FURL procedure. Furthermore, we highly recommend that a ureteral stent should be placed prior to FURL in patients with SII > 1,300 and one of the following criteria: age > 60 years, urinary white blood cell levels of 1+/2+/3+, or positive urine bacterial culture (notably, approximately 80% of patients in this study developed SIRS).

However, there are still some limitations in our study. Firstly, the restricted sample size may introduce a potential bias in our results. Secondly, some variables, such as gender, diabetes, stone size, ureteral access sheath, and surgery time, did not demonstrate statistical significance in this study, but they have been recognized as influencing the risk of SIRS in existing literature (6, 30, 31). Lastly, this study is a retrospective analysis conducted within a single medical institution and requires further prospective studies and external validation to corroborate our results.

## 5 Conclusions

This study established a convenient nomogram incorporating age, urine WBC, urine bacterial culture, and SII value to preoperatively predict the risk of SIRS in patients undergoing one-stage FURL. Internal validation demonstrated that the nomogram has favorable predictive capability and clinical applicability. We are the first to introduce a risk scoring system for SIRS in patients undergoing one-stage FURL, identifying SII as the most relevant risk factor. We strongly recommend the placement of a ureteral stent prior to FURL in patients with SII > 1,300 and meeting one of the following: age > 60 years, urinary white blood cell levels of 1+/2+/3+, or positive urine bacterial culture. These novel insights may assist clinicians in selecting safer therapeutic approaches for UUTS patients.

## Data Availability

The original contributions presented in the study are included in the article/Supplementary Material, further inquiries can be directed to the corresponding author.

## References

[1] Ma YC Jian ZY Li H Wang K. Preoperative urine nitrite versus urine culture for predicting postoperative fever following flexible ureteroscopic lithotripsy: a propensity score matching analysis. World J Urol. (2021) 39(3):897–905. 10.1007/s00345-020-03240-w 32430571

[2] Ma C Lu J Zhu YY Huo YJ Xia SJ Shao Y. Systemic inflammatory response syndrome combined with pre- and postoperative white blood cell ratio is a better criterion to identify septic shock patients after flexible ureteroscopic lithotripsy. J Endourol. (2021) 35(7):973–78. 10.1089/end.2020.1002 33218256

[3] Assimos D Crisci A Culkin D Xue W Roelofs A Duvdevani M Preoperative JJ stent placement in ureteric and renal stone treatment: results from the Clinical Research Office of Endourological Society (CROES) ureteroscopy (URS) global study. BJU Int. (2016) 117(4):648–54. 10.1111/bju.13250 26237735

[4] Huang WN Huang HL Wang YH Chen WX Deng H Zhong MZ. Application of 11/13Fr suctioning ureteral access sheath and 8.55Fr single-use digital flexible ureteroscope in one-stage flexible ureteroscopic lithotripsy: an initial experience of 900 cases. Urolithiasis. (2024) 52(1):112. 10.1007/s00240-024-01607-x 39105853

[5] Pan Y Chen H Chen HL Jin XX Zhu YX Chen G. The feasibility of one-stage flexible ureteroscopy lithotripsy in solitary kidney patients with 1–3 cm renal stones and risk factors of renal function changes. Ren Fail. (2021) 43(1):264–72. 10.1080/0886022X.2021.1872625 33491554 PMC7850451

[6] Mi QW Meng XJ Meng LH Chen D Fang SW. Risk factors for systemic inflammatory response syndrome induced by flexible ureteroscope combined with holmium laser lithotripsy. Biomed Res Int. (2020) 2020:6842479. 10.1155/2020/6842479 32280696 PMC7128057

[7] Wang LB Yu XZ Qiu ZZ Liu PY Tian W He W Influence of preoperative urine culture and bacterial species on urogenital sepsis after ureteral flexible lithotripsy in patients with upper urinary tract stones. Front Med (Lausanne). (2024) 11:1393734. 10.3389/fmed.2024.1393734 38765255 PMC11099900

[8] Levy M Artigas A Phillips G Rhodes A Beale R Osborn T Outcomes of the Surviving Sepsis Campaign in intensive care units in the USA and Europe: a prospective cohort study. Lancet Infect Dis. (2012) 12(12):919–24. 10.1016/S1473-3099(12)70239-6 23103175

[9] Schefzik R Hahn B Schneider LV. Dissecting contributions of individual systemic inflammatory response syndrome criteria from a prospective algorithm to the prediction and diagnosis of sepsis in a polytrauma cohort. Front Med (Lausanne). (2023) 31(10):1227031. 10.3389/fmed.2023.1227031 PMC10424878 37583420

[10] Ye JJ Hu XD Wang ZW Li R Gan L Zhang MW The role of mtDAMPs in the trauma-induced systemic inflammatory response syndrome. Front Immunol. (2023) 18(14):1164187. 10.3389/fimmu.2023.1164187 PMC10391641 37533869

[11] Wu WJ Zhang D Jin TT Lu TY Zhou FH. Progress in the study of biomarkers for early prediction of systemic inflammatory response syndrome after percutaneous nephrolithotomy. Front Immunol. (2023) 30(14):1142346. 10.3389/fmed.2023.1227031 PMC10097887 37063849

[12] Askim A Moser F Gustad L Stene H Gundersen M Åsvold B Poor performance of quick-SOFA (qSOFA) score in predicting severe sepsis and mortality—a prospective study of patients admitted with infection to the emergency department. Scand J Trauma Resusc Emerg Med. (2017) 25(1):56. 10.1186/s13049-017-0399-4 28599661 PMC5466747

[13] Evans L Rhodes A Alhazzani W Antonelli M Coopersmith CM French C Surviving sepsis campaign: international guidelines for management of sepsis and septic shock 2021. Intensive Care Med. (2021) 47(11):1181–247. 10.1097/CCM.0000000000005337 34599691 PMC8486643

[14] Xu P Zhang SK Zhang YY Zeng T Chen D Wu WZ Enhanced antibiotic treatment based on positive urine dipstick infection test before percutaneous nephrolithotomy did not prevent postoperative infection in patients with negative urine culture. J Endourol. (2021) 35(12):1743–9. 10.1089/end.2021.0185 34002622

[15] Pricop M Ancusa O Talpos S Urechescu H Bumbu BA. The predictive value of systemic immune-inflammation index and symptom severity score for sepsis and systemic inflammatory response syndrome in odontogenic infections. J Pers Med. (2022) 12(12):2026. 10.3390/jpm12122026 36556246 PMC9782876

[16] Staniewska E Grudzien K Stankiewicz M Raczek ZK Rembak SJ Nowicka Z The prognostic value of the systemic immune-inflammation index (SII) and red cell distribution width (RDW) in patients with cervical cancer treated using radiotherapy. Cancers (Basel). (2024) 16(8):1542. 10.3390/cancers16081542 38672624 PMC11049631

[17] Guo WC Song YC Sun Y Du HS Cai Y You QQ Systemic immune-inflammation index is associated with diabetic kidney disease in type 2 diabetes mellitus patients: evidence from NHANES 2011–2018. Front Endocrinol (Lausanne). (2022) 6(13):1071465. 10.3389/fendo.2022.1071465 PMC9763451 36561561

[18] Zhang HS Lin J Huang YF Chen Y. The systemic immune-inflammation index as an independent predictor of survival in patients with locally advanced esophageal squamous cell carcinoma undergoing neoadjuvant radiotherapy. J Inflamm Res. (2024) 11(17):4575–86. 10.2147/JIR.S463163 PMC11249110 39011418

[19] Telang S Mayfield CK Palmer R Liu KC Wier J Hong K Preoperative laboratory values predicting periprosthetic joint infection in morbidly obese patients undergoing total hip or knee arthroplasty. J Bone Joint Surg Am. (2024) 106(14):1317–27. 10.2106/JBJS.23.01360 38941451

[20] Xu PP Cao Y Ren RQ Zhang S Zhang C Hao PP Usefulness of the systemic inflammation response Index and the systemic immune inflammation index in predicting restenosis after stent implantation. J Inflamm Res. (2024) 23(17):4941–55. 10.2147/JIR.S461277 PMC11268991 39051057

[21] Miao HH Ge DY Wang QW Zhou LL Chen HS Qin YB Predictive significance of systemic immune-inflammation index combined with prealbumin for postoperative pneumonia following lung resection surgery. BMC Pulm Med. (2024) 24(1):277. 10.1186/s12890-024-03086-7 38862955 PMC11167804

[22] Wang SY Wan Y Zhang WB. The clinical value of systemic immune inflammation index (SII) in predicting the severity of hospitalized children with Mycoplasma Pneumoniae pneumonia: a retrospective study. Int J Gen Med. (2024) 17:935–42. 10.2147/IJGM.S451466 38495920 PMC10944171

[23] Peng C Li JL Xu G Jin J Chen JJ Pan SH. Significance of preoperative systemic immune-inflammation (SII) in predicting postoperative systemic inflammatory response syndrome after percutaneous nephrolithotomy. Urolithiasis. (2021) 49(6):513–9. 10.1007/s00240-021-01266-2 33835228

[24] Chen D Jiang CH Liang XF Zhong FL Huang J Lin YP Early and rapid prediction of postoperative infections following percutaneous nephrolithotomy in patients with complex kidney stones. BJU Int. (2019) 123(6):1041–7. 10.1111/bju.14484 30007112

[25] Benson AD Juliano TM Miller NL. Infectious outcomes of nephrostomy drainage before percutaneous nephrolithotomy compared with concurrent access. J Urol. (2014) 192(3):770–4. 10.1016/j.juro.2014.03.004 24631102

[26] Leon MD Lundsberg LS Culhane J Zhang J Son M Reddy UM. Fetal growth restriction and small for gestational age as predictors of neonatal morbidity: which growth nomogram to use? Am J Obstet Gynecol. (2023) 229(6):678. 10.1016/j.ajog.2023.06.035 37348779

[27] Zhou Y Lin CM Zhu L Zhang RT Cheng L Chang YY. Nomograms and scoring system for forecasting overall and cancer-specific survival of patients with prostate cancer. Cancer Med. (2023) 12(3):2600–13. 10.1002/cam4.5137 35993499 PMC9939188

[28] Sun JX Xu JZ Liu CQ Xun Y Lu JL Xu MY. A novel nomogram for predicting post-operative sepsis for patients with solitary, unilateral and proximal ureteral stones after treatment using percutaneous nephrolithotomy or flexible ureteroscopy. Front Surg. (2022) 15(9):814293. 10.3389/fsurg.2022.814293 PMC9051077 35495750

[29] Xuan ZJ Yu ZK Tan GB Ding N He HB Yu SC Development and validation of a novel nomogram for predicting systemic inflammatory response syndrome’s occurrence in patients undertaking flexible ureteroscopy. Transl Androl Urol. (2022) 11(2):228–37. 10.21037/tau-22-34 35280653 PMC8899144

[30] Giulioni C Brocca C Gauhar V Somani BK Chew BH Traxe O Does age impact outcomes of retrograde intrarenal surgery in the elderly? Results from 366 patients from the FLEXible ureteroscopy outcomes registry (FLEXOR). Aging Clin Exp Res. (2023) 35(11):2711–19. 10.1007/s40520-023-02545-1 37682489 PMC10627914

[31] Mohanty S Kamolvit W Scheffschick A Björklund A Tovi J Espinosa A Diabetes downregulates the antimicrobial peptide psoriasin and increases E. coli burden in the urinary bladder. Nat Commun. (2022) 13(1):4983. 10.1038/s41467-022-32636-y 36127330 PMC9489794

